# Comorbidity network characteristics of depressive and anxiety symptoms and their associations with quality of life in patients with spinal cord injury: a cross-sectional study

**DOI:** 10.3389/fpsyt.2026.1861743

**Published:** 2026-07-24

**Authors:** Lu Lu, Xinyuan Qiu, Yiqi Lai, Jia Ye, Mingzhu Fang, Shengli Ma, Pengkun Xue, Sihan Meng, Chaoqun Guo, Zhe Li, Rongbin Chen

**Affiliations:** 1 Department of Rehabilitation, The First Affiliated Hospital, Fujian Medical University, Fuzhou, China; 2 Department of Rehabilitation, National Regional Medical Center, the First Affiliated Hospital, Fujian Medical University, Fuzhou, China; 3 Department of Rehabilitation Medicine, The Fifth Affiliated Hospital of Zhengzhou University, Zhengzhou, China; 4 Department of Pulmonary and Critical Care Medicine, 900th Hospital of PLA Joint Logistic Support Force, Fuzhou, China; 5 Department of Spinal Cord Injury, Shaanxi Rehabilitation Hospital, Xi’an, China

**Keywords:** anxiety, bridge symptoms, core symptoms, depression, quality of life, spinal cord injury, symptom network analysis

## Abstract

**Objective:**

To examine the topological structure of the comorbidity network of depressive and anxiety symptoms in patients with subacute spinal cord injury (SCI), identify core and bridge symptoms, and explore the independent associations between specific symptoms and quality-of-life outcomes.

**Methods:**

A total of 316 patients with subacute SCI were assessed using the Patient Health Questionnaire-9 (PHQ-9), the Generalized Anxiety Disorder-7 (GAD-7), and the World Health Organization Quality of Life Brief Version (WHOQOL-BREF). A Gaussian graphical model (GGM) was estimated to construct the depression–anxiety symptom network. Expected influence (EI) and bridge expected influence (bEI) were calculated to identify core and bridge nodes. Flow network analyses were performed to evaluate independent conditional associations between symptoms and quality-of-life outcomes. Network comparison tests (NCTs) were used to examine network invariance across key clinical subgroups. Ordinal PHQ-9 and GAD-7 items were handled using cor_auto-derived polychoric correlations where appropriate, and sensitivity analyses examined EBIC tuning, no-jitter estimation, data-driven communities, and covariate-adjusted flow networks.

**Results:**

Centrality analyses showed that guilt (PHQ.6, EI = 1.257), irritability (GAD.6, EI = 1.096), and psychomotor changes (PHQ.8, EI = 1.006) were among the most influential symptoms in the network, whereas depressed mood (PHQ.2, bEI = 1.241), concentration difficulties (PHQ.7, bEI = 0.972), irritability (GAD.6, bEI = 0.954), and psychomotor changes (PHQ.8, bEI = 0.942) showed the greatest bridge effects across the depression and anxiety communities. The strongest edges in the network were PHQ.9–PHQ.8 (weight = 0.396), PHQ.5–PHQ.4 (weight = 0.395), and PHQ.8–GAD.5 (weight = 0.384). Flow network analyses indicated that suicidal ideation, depressed mood, and restlessness were more strongly negatively associated with general health, whereas concentration difficulties and psychomotor changes were more strongly negatively associated with overall quality of life. NCT results showed no significant differences in network structure or global strength across sex or injury severity subgroups (P > 0.05). Bootstrap analyses supported acceptable network stability. All bivariate symptom-QOL correlations were negative after verification of scoring direction; positive symptom-QOL partial edges were therefore interpreted cautiously as conditional suppression effects rather than as evidence that symptoms were associated with better QOL.

**Conclusion:**

The depression–anxiety comorbidity network in patients with SCI appears to be organized around a limited number of highly influential symptoms, particularly guilt, irritability, and psychomotor abnormalities. Moving beyond total questionnaire scores, prioritizing symptoms with high centrality, strong bridge effects, and close links to quality-of-life outcomes may help refine psychological assessment and intervention planning in SCI rehabilitation. All edges should be interpreted as undirected conditional associations, not causal pathways.

## 1 Introduction

Spinal cord injury (SCI) is a severe central nervous system disorder caused by traumatic or non-traumatic etiologies and is commonly associated with complete or partial loss of sensory, motor, and autonomic function below the level of injury. With continuing advances in acute care and rehabilitation, survival after SCI has improved substantially; however, lifelong physical disability, altered social roles, and prolonged adaptation challenges impose a considerable psychosocial burden on affected individuals and markedly compromise quality of life (1, 2). Among the psychological sequelae of SCI, depression and anxiety are the most prevalent emotional problems. Meta-analytic evidence suggests that depressive symptoms are highly prevalent in people with SCI, while anxiety is also common, and the two often co-occur (3, 4). This high level of comorbidity not only intensifies subjective suffering but is also associated with poorer functional outcomes, lower rehabilitation adherence, and less favorable long-term psychological adjustment (5).

Most previous studies on emotional problems in SCI have relied on latent-variable approaches and have used total scores to quantify depression and anxiety severity. Although total-score models are useful for summarizing overall symptom burden, they obscure the complex interactions among individual symptoms within these heterogeneous syndromes and provide limited insight into the specific roles of particular symptoms in maintaining comorbidity (6). Consequently, interventions derived from such models may fail to identify the most clinically relevant targets for individualized psychological management.

In recent years, symptom network analysis has emerged as a useful framework for understanding the internal structure of mental disorders. According to network theory, mental disorders do not necessarily arise from a single underlying latent entity; instead, they may reflect a system of mutually interacting symptoms linked through conditional dependence relations (7, 8). Within this framework, expected influence (EI) can be used to identify “core symptoms” that exert relatively large global influence on the overall symptom network (9), whereas bridge centrality—especially bridge expected influence (bEI)—can be used to identify “bridge symptoms” that connect different symptom communities and may facilitate the spread of comorbidity (10). Identifying such core and bridge symptoms may help clarify the micro-level architecture of depression–anxiety comorbidity and inform more targeted clinical interventions.

Quality of life (QOL) is a key endpoint in SCI rehabilitation and encompasses multiple domains, including physical health, psychological well-being, social relationships, and global subjective health. Prior studies have shown that depression and anxiety are not only highly intertwined but are also negatively associated with QOL impairment (11–13). Integrating QOL as an external outcome into a symptom network framework may help determine which specific emotional symptoms are most directly linked to subjective health and overall life quality, thereby moving beyond a simple “reduce total symptom scores” approach and identifying symptom-level targets that are more strongly related to patients’ lived experiences.

Against this background, evidence remains limited regarding the fine-grained symptom network structure of depression–anxiety comorbidity in SCI. Therefore, this cross-sectional study aimed to: (1) construct the comorbidity network of depressive and anxiety symptoms in patients with subacute SCI; (2) calculate EI and bEI to identify core symptoms that sustain the network and bridge symptoms that link the depression and anxiety communities; and (3) explore the independent statistical associations between specific symptom nodes and QOL outcomes. We expected these findings to provide methodological and empirical support for more precise psychological screening, symptom-targeted rehabilitation planning, and QOL-oriented management in SCI.

## 2 Methods

### 2.1 Study design and ethical approval

This study used a cross-sectional observational design. Participants were patients with SCI who received rehabilitation treatment at the Fifth Affiliated Hospital of Zhengzhou University between June 2023 and June 2025. The study was conducted in accordance with the Declaration of Helsinki and was approved by the Ethics Committee of the Fifth Affiliated Hospital of Zhengzhou University (KY2023003). Written informed consent was obtained from all participants or their legal guardians. Because cross-sectional network analysis is designed to characterize conditional dependence structures among variables rather than establish temporal order or causal direction, the present study focused on describing network topology, identifying key nodes, and examining statistical associations with quality-of-life outcomes, without making causal inferences.

### 2.2 Participants and eligibility criteria

The inclusion criteria were: (1) a clinically confirmed first episode of SCI; (2) age 18–80 years; (3) disease duration ≤ 6 months, corresponding to the subacute rehabilitation stage; (4) sufficient language comprehension and expression to complete the assessment; and (5) voluntary participation with written informed consent. The exclusion criteria were: (1) prominent aphasia, severe cognitive impairment, or other conditions precluding valid completion of the assessments; and (2) substantial missing questionnaire data that prevented statistical analysis.

A total of 316 eligible patients were included, comprising 262 men (82.9%) and 54 women (17.1%). The mean age was 40.57 ± 11.75 years, and the mean time since injury was 3.34 ± 1.38 months. Injury levels were cervical in 144 patients (45.6%), thoracic in 113 (35.8%), and lumbosacral in 59 (18.7%). According to the ASIA Impairment Scale (AIS), 100 patients (31.6%) had complete injury (AIS A) and 216 (68.4%) had incomplete injury (AIS B–D).

### 2.3 Measures

#### 2.3.1 Depressive symptoms

Depressive symptoms were assessed using the Patient Health Questionnaire-9 (PHQ-9), which measures the frequency of depressive symptoms during the preceding 2 weeks. The scale includes 9 items rated from 0 (“not at all”) to 3 (“nearly every day”). The Chinese version of the PHQ-9 has shown good reliability and validity (14). To avoid obscuring symptom-level heterogeneity, the 9 individual PHQ-9 items were entered directly into the network analysis as depressive symptom nodes (6, 15).

#### 2.3.2 Anxiety symptoms

Anxiety symptoms were assessed using the Generalized Anxiety Disorder-7 (GAD-7), a 7-item scale measuring core anxiety symptoms over the previous 2 weeks (16). Each item is rated using the same 0–3 response format as the PHQ-9. The 7 individual GAD-7 items were entered directly into the network model.

#### 2.3.3 Quality of life

Quality of life was assessed using the Chinese version of the World Health Organization Quality of Life Brief Version (WHOQOL-BREF) (17). The present analysis used the two global WHOQOL-BREF items: overall quality of life and general health. Both items are positively scaled from 1 to 5, with higher scores indicating better perceived quality of life or health satisfaction; no reverse scoring was applied to these two global items. A two-item global QOL score was calculated by summing the two items (range 2–10) for comparability with the original analysis. Because these two WHOQOL-BREF global items are recommended to be interpreted as separate single-item indicators and did not show adequate internal consistency in this cohort, item-specific flow analyses for overall QOL and general health were emphasized, whereas the composite QOL node was treated as an exploratory sensitivity outcome. Standardization was used only for visualization of centrality indices and did not change the direction of item scaling.

### 2.4 Statistical analysis and network modeling

#### 2.4.1 Descriptive statistics

Descriptive analyses were performed using standard statistical procedures. Normally distributed continuous variables were summarized as mean ± standard deviation, non-normally distributed variables as median and interquartile range, and categorical variables as frequency and percentage. For each symptom item, the mean, standard deviation, skewness, and kurtosis were also calculated. Item-level missingness was inspected before network estimation. Probable depression and anxiety were summarized using standard PHQ-9 and GAD-7 cutoffs of ≥10, and mild-or-greater symptom burden was additionally reported using the ≥5 threshold to facilitate comparison with screening-oriented SCI literature.

#### 2.4.2 Network estimation

A Gaussian graphical model (GGM) was estimated in R based on the 16 symptom items from the PHQ-9 and GAD-7 to characterize the depression–anxiety comorbidity network. All PHQ-9 and GAD-7 items are four-category ordinal variables; therefore, the correlation matrix was estimated with cor_auto within the bootnet/qgraph framework, which identifies ordinal variables and estimates polychoric correlations where appropriate. Next, a regularized partial correlation network was estimated using the graphical least absolute shrinkage and selection operator with the extended Bayesian information criterion (EBICglasso), which yields a sparse and interpretable network structure (8). The EBIC tuning parameter γ was set to 0.5 because this commonly used conservative setting balances sensitivity and specificity in psychological network estimation and reduces the risk of retaining spurious edges (18). To evaluate dependence on this tuning choice, the analyses were repeated with γ = 0.25 and γ = 0.75. Because some low-frequency items can induce near-zero variance or non-positive definite covariance matrices during bootstrap resampling, a very small Gaussian random perturbation (mean = 0, standard deviation = 1 × 10−6) was added to the raw item-level data before the primary estimation. This perturbation was several orders of magnitude smaller than one response category on the 0–3 symptom scales and was used solely to avoid numerical instability. Robustness was checked by re-estimating networks without this perturbation using Pearson, Spearman, and polychoric partial-correlation approaches. In the network, nodes represent individual symptoms, and edges represent conditional dependence relations between pairs of symptoms after controlling for all other nodes in the network; thicker edges indicate stronger associations.

#### 2.4.3 Core symptoms, bridge symptoms, and node predictability

Expected influence (EI) was used to quantify node centrality. By summing edge weights algebraically, EI can more robustly capture the global influence of a node in networks that include negative edges (9). Bridge expected influence (bEI) was used to quantify the extent to which nodes connect the depression and anxiety communities, thereby identifying symptoms with potential bridge roles in comorbidity (10). The primary bridge analysis used the theoretically defined scale communities (PHQ-9 depression items vs. GAD-7 anxiety items). As a robustness check, a modularity-based data-driven community-detection analysis (greedy modularity algorithm applied to absolute regularized edge weights) was performed to evaluate whether the empirical symptom structure recovered the same community partition. In addition, node predictability was estimated using the mgm package and expressed as R2, representing the proportion of variance in a given symptom node explained jointly by its neighboring nodes. This index reflects the degree to which a node is statistically predictable from the local network and may offer information on the potential malleability of symptoms (19). Numerical R2 values for all nodes are reported in **Supplementary Table 3**. All centrality indices were transformed into z scores for visualization.

#### 2.4.4 Network accuracy and stability

The accuracy and stability of the network were evaluated using the bootnet package (8). First, 95% confidence intervals (CIs) for edge weights were estimated using 1,000 nonparametric bootstrap resamples. Second, case-dropping bootstrap procedures were used to assess the stability of centrality indices, and the correlation stability coefficient (CS-coefficient) was calculated. A CS-coefficient > 0.25 is generally considered acceptable, whereas a value > 0.50 indicates good stability of centrality ranking. Bootstrap difference tests (α = 0.05) were also used to compare edge weights and node centrality values. We additionally examined whether the negative PHQ.6–GAD.1 edge and the low EI ranking of PHQ.9 were reproduced in no-jitter sensitivity analyses to evaluate possible suppression artifacts.

#### 2.4.5 Flow network analysis

To examine independent statistical associations between emotional symptoms and quality-of-life outcomes, flow network analyses were conducted. For each flow network, an augmented network containing the 16 symptom nodes and one QOL node was estimated using the same regularization settings as the primary network (cor_auto-derived correlations and EBICglasso with γ = 0.5), and the edges adjacent to the QOL node were then extracted. Thus, flow edges represent undirected conditional associations after adjustment for all other nodes in the network, not directional or causal effects. In supplementary analyses, the same procedure was repeated with “general health” and “overall quality of life” as external outcome nodes. Positive and negative edges indicate positive and negative independent conditional associations between symptoms and outcomes, and the absolute edge weight reflects association strength. Because higher PHQ-9/GAD-7 scores indicate greater symptom severity whereas higher WHOQOL-BREF scores indicate better QOL, all signs were interpreted only after verifying the scaling direction and examining bivariate symptom-QOL correlations. As a covariate-adjusted sensitivity analysis, flow associations were re-estimated after residualizing symptoms and QOL variables for age, sex, time since injury, injury segment, and AIS severity. Pain severity, psychotropic medication, and external sleep-quality measures were not available in the dataset and were therefore addressed as limitations rather than adjusted covariates.

#### 2.4.6 Subgroup comparisons

Subgroup analyses were conducted by sex (male vs. female) and injury severity (complete vs. incomplete SCI) using the Network Comparison Test (NCT) (20). Based on 1,000 permutation iterations, we tested network structure invariance and global strength invariance between subgroups. When results did not reach statistical significance (*P* > 0.05), they were conservatively interpreted as indicating that no statistically significant difference was detected.

## 3 Results

### 3.1 Sample characteristics and descriptive symptom statistics

A total of 316 patients with SCI who met the eligibility criteria were included in the analysis (**Table 1**). Of these, 262 were male (82.9%) and 54 were female (17.1%). The mean age was 40.57 ± 11.75 years, and the mean time since injury was 3.34 ± 1.38 months. Injury levels were cervical in 144 patients (45.6%), thoracic in 113 (35.8%), and lumbosacral in 59 (18.7%). According to the ASIA Impairment Scale, 100 patients (31.6%) had complete injury (AIS A), whereas 216 (68.4%) had incomplete injury (AIS B–D). All 316 participants had complete item-level data for the PHQ-9, GAD-7, WHOQOL-BREF global items, and recorded clinical variables; therefore, no imputation was performed. Using the conventional ≥10 cutoffs, 27 patients (8.5%) screened positive for probable depression and 14 patients (4.4%) screened positive for probable anxiety; 11 patients (3.5%) met both cutoffs. Using the ≥5 threshold for mild-or-greater symptoms, 111 patients (35.1%) screened positive for depression, 103 (32.6%) screened positive for anxiety, and 85 (26.9%) met both thresholds. Descriptive statistics for the 16 depressive and anxiety symptom items are shown in **Supplementary Table 1**. The four items with the highest mean scores were PHQ.4 (fatigue, 0.70 ± 0.70), GAD.1 (nervousness, 0.63 ± 0.62), PHQ.3 (sleep problems, 0.59 ± 0.71), and GAD.6 (irritability, 0.57 ± 0.63). The item with the lowest mean score was PHQ.9 (suicidal ideation, 0.14 ± 0.39). In terms of distributional characteristics, PHQ.9 showed the highest skewness (3.16) and kurtosis (12.26), indicating that high-end responses were relatively rare in this sample. The PHQ-9 and GAD-7 showed high internal consistency (Cronbach’s α = 0.919 and 0.921, respectively). The two WHOQOL-BREF global items were not treated as an internally consistent scale in this sample (Pearson r = −0.397; Cronbach’s α = −1.318), supporting the revised emphasis on item-specific QOL analyses. These screening rates should be interpreted in light of prior SCI meta-analytic estimates: the ≥10 probable-depression rate in this subacute rehabilitation cohort was lower than the reported pooled depression estimate of approximately 22.2%, whereas the ≥5 mild-or-greater depression/anxiety burden indicated that clinically relevant symptoms remained common. Likewise, the GAD-7 ≥10 probable-anxiety rate was lower than the approximately 27% self-reported clinically significant anxiety estimate reported in SCI meta-analytic literature, while the ≥5 anxiety burden was closer to broad screening-based caseness.

**Table 1:** Baseline sociodemographic and clinical characteristics of patients with spinal cord injury.

Variable	Level/summary	n	%
Gender	Female	54	17.10%
	Male	262	82.90%
Age	average	40.57 ± 11.75	
	18-40	159	50.30%
	41-60	138	43.70%
	>60	19	6.00%
Time since injury (months)	average	3.34 ± 1.38	
	1-2	92	29.10%
	3-4	151	47.80%
	5-6	73	23.10%
Injured part	Cervical	144	45.60%
	Thoracic	113	35.80%
	Lumbar/Sacral	59	18.70%
Injury severity (AIS)	AIS-A	100	31.60%
	AIS-B	45	14.20%
	AIS-C	70	22.20%
	AIS-D	101	32.00%

Age and time since injury are presented as mean ± standard deviation; all other values are shown as counts and percentages. AIS, American Spinal Injury Association Impairment Scale.

### 3.2 Depression–anxiety comorbidity network structure

**Figure 1:**
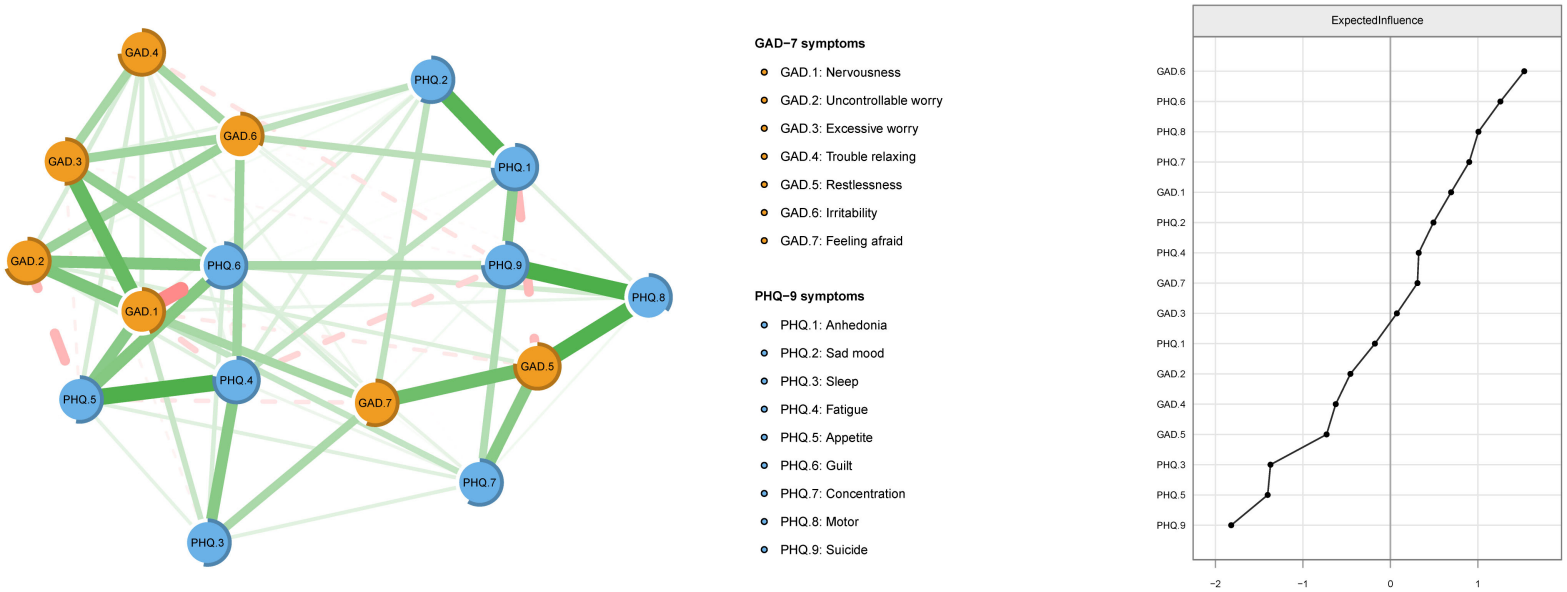
Network structure of comorbid depressive and anxiety symptoms and node expected influence in patients with spinal cord injury. The left panel shows the depression–anxiety symptom network constructed from the 16 PHQ-9 and GAD-7 items. Nodes represent specific symptoms, with blue nodes indicating depressive symptoms and orange nodes indicating anxiety symptoms. Edges represent regularized partial correlations estimated using the EBICglasso model; green solid lines indicate positive associations and pink dashed lines indicate negative associations. Edge thickness and color saturation reflect association strength. The dark ring surrounding each node indicates node predictability, defined as the proportion of node variance explained by all neighboring nodes. The right panel displays the ranking of node expected influence (EI). Higher EI values indicate greater global influence of the node within the overall symptom network. PHQ-9, Patient Health Questionnaire-9; GAD-7, Generalized Anxiety Disorder-7; EI, expected influence.

The EBICglasso-regularized partial correlation network based on the 16 PHQ-9 and GAD-7 items is presented in **Figure 1**. The overall network was dominated by positive partial correlation edges, although a small number of negative edges were also observed. Complete edge-weight information is provided in **Supplementary Table 2**. Across all edges, the five strongest independent partial correlations were PHQ.9 (suicidal ideation)–PHQ.8 (psychomotor changes) (weight = 0.396), PHQ.5 (appetite changes)–PHQ.4 (fatigue) (weight = 0.395), PHQ.8 (psychomotor changes)–GAD.5 (restlessness) (weight = 0.384), PHQ.2 (depressed mood)–PHQ.1 (anhedonia) (weight = 0.371), and GAD.3 (excessive worry)–GAD.1 (nervousness) (weight = 0.346). The strongest negative partial correlation in the network was observed between PHQ.6 (guilt) and GAD.1 (nervousness) (weight = -0.364). Conceptually, the PHQ.9–PHQ.8 edge links suicidal ideation with psychomotor disturbance, the PHQ.5–PHQ.4 edge links appetite change with fatigue, and the PHQ.8–GAD.5 edge reflects overlapping behavioral activation/restlessness content across depression and anxiety. The PHQ.2–PHQ.1 and GAD.3–GAD.1 edges connect core affective and worry-related contents within their respective symptom domains. The negative PHQ.6–GAD.1 edge was not interpreted as a protective relation; rather, it was evaluated as a possible suppression effect in sensitivity analyses.

### 3.3 Core and bridge nodes

**Figure 2:**
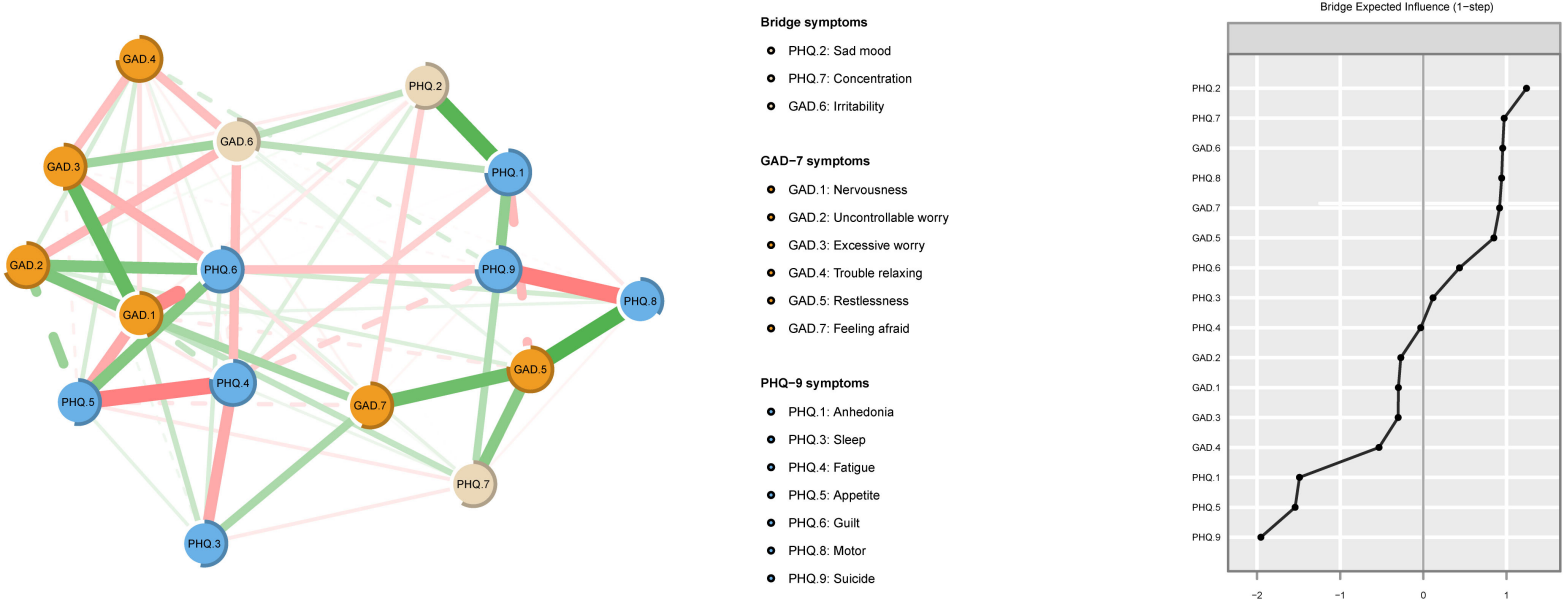
Bridge structure of the depression–anxiety symptom network and bridge expected influence. The left panel shows the bridge structure between the depressive and anxiety symptom communities; light-brown nodes indicate symptoms with relatively stronger bridge roles. Edges represent regularized partial correlations; green solid lines indicate positive associations and pink dashed lines indicate negative associations, with thicker lines reflecting larger absolute edge weights. The right panel shows the ranking of bridge expected influence [bEI (1-step)]. Higher bEI values indicate a stronger role of the node in linking the depression and anxiety symptom communities. PHQ-9, Patient Health Questionnaire-9; GAD-7, Generalized Anxiety Disorder-7; bEI, bridge expected influence.

The centrality and bridge-centrality results are shown in **Figure 2**. In terms of expected influence (EI), the four highest-ranking symptoms were PHQ.6 (guilt, EI = 1.257), GAD.6 (irritability, EI = 1.096), PHQ.8 (psychomotor changes, EI = 1.006), and PHQ.7 (concentration difficulties, EI = 0.901). The three nodes with the lowest EI values were PHQ.9 (suicidal ideation, EI = -1.820), PHQ.5 (appetite changes, EI = -1.403), and PHQ.3 (sleep problems, EI = -1.371). In terms of bridge expected influence (bEI), the four highest-ranking nodes were PHQ.2 (depressed mood, bEI = 1.241), PHQ.7 (concentration difficulties, bEI = 0.972), GAD.6 (irritability, bEI = 0.954), and PHQ.8 (psychomotor changes, bEI = 0.942). In contrast, PHQ.9 (suicidal ideation) had the lowest bEI value (-1.954). Node predictability is displayed as dark outer rings around nodes in **Figure 1**. The data-driven community analysis identified three mixed communities rather than a strict PHQ-versus-GAD partition: (1) GAD.1, GAD.2, GAD.3, GAD.4, PHQ.3, PHQ.4, PHQ.5, and PHQ.6; (2) GAD.5, GAD.7, PHQ.7, PHQ.8, and PHQ.9; and (3) GAD.6, PHQ.1, and PHQ.2. The adjusted Rand index comparing this empirical solution with the PHQ/GAD scale partition was -0.084, indicating weak alignment. Therefore, the primary bEI results should be understood as scale-defined bridge centrality between depression and anxiety, while the data-driven community results support a clinically mixed affective-somatic topology. In the data-driven bridge analysis, GAD.6, GAD.7, PHQ.7, and PHQ.6 remained among the strongest cross-community nodes. Sensitivity analyses without jitter showed broadly similar high-ranking nodes and reproduced the negative PHQ.6-GAD.1 edge in dense partial-correlation checks (Pearson partial r = -0.148, Spearman partial r = -0.166, polychoric partial r = -0.682), although this edge was interpreted cautiously because its magnitude varied across methods (**Supplementary Table 4**).

### 3.4 Network accuracy and stability

**Figure 3:**
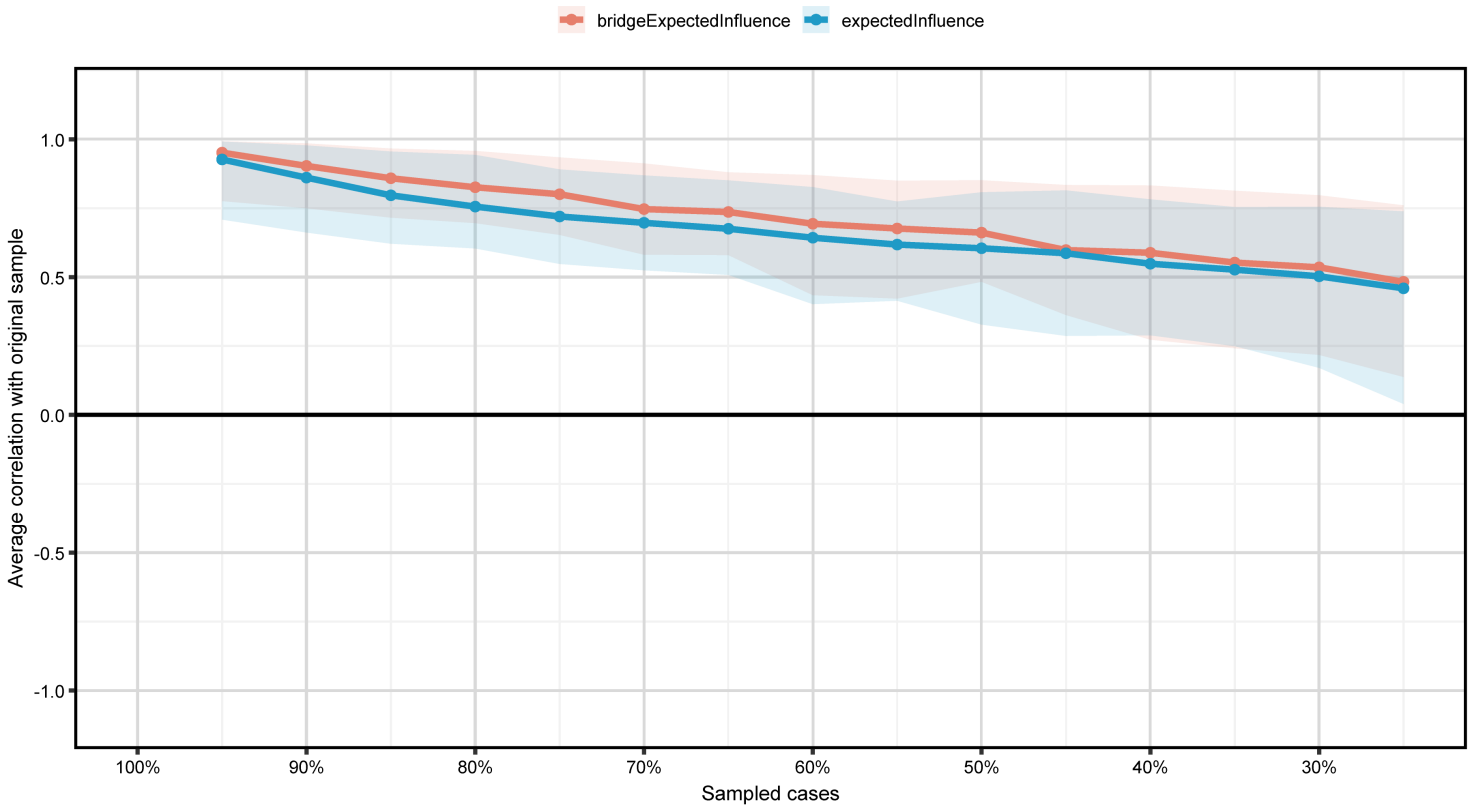
Case-dropping bootstrap assessment of centrality stability. This figure shows the mean correlations between the centrality indices re-estimated after progressively dropping portions of the sample and the corresponding indices in the original sample. The horizontal axis indicates the proportion of the sample retained, and the vertical axis indicates the mean correlation with the original centrality estimates. The red line represents bridge expected influence, and the blue line represents expected influence; shaded regions indicate uncertainty around the estimates. This image was used to evaluate the stability of the centrality indices under sample perturbation.

The results of network accuracy and stability analyses are presented in **Figure 3** and **Supplementary Figures 1, 2**. The nonparametric bootstrap analysis based on 1,000 resamples showed relatively narrow 95% confidence intervals for the main edge weights, and most of these intervals did not cross zero (**Supplementary Figure 1**). The bootstrap edge-difference test (**Supplementary Figure 2**) indicated that some of the highest-weight edges were significantly stronger than many low- to moderate-weight edges in the network (P < 0.05). Case-dropping bootstrap results showed that when 95% of the sample was retained, the mean correlations between the re-estimated and original networks were 0.952 for bEI and 0.927 for EI. Even after 50% of the sample had been dropped, the corresponding mean correlations remained 0.661 for bEI and 0.605 for EI (**Figure 3**). The CS-coefficients were 0.438 for bEI and 0.361 for EI, exceeding the commonly recommended acceptability threshold of 0.25. Numerical node predictability values are reported in **Supplementary Table 3**; R2 ranged from 0.437 (PHQ.9) to 0.702 (GAD.6), with a mean of 0.621.

### 3.5 Flow network associations with quality of life

**Figure 4:**
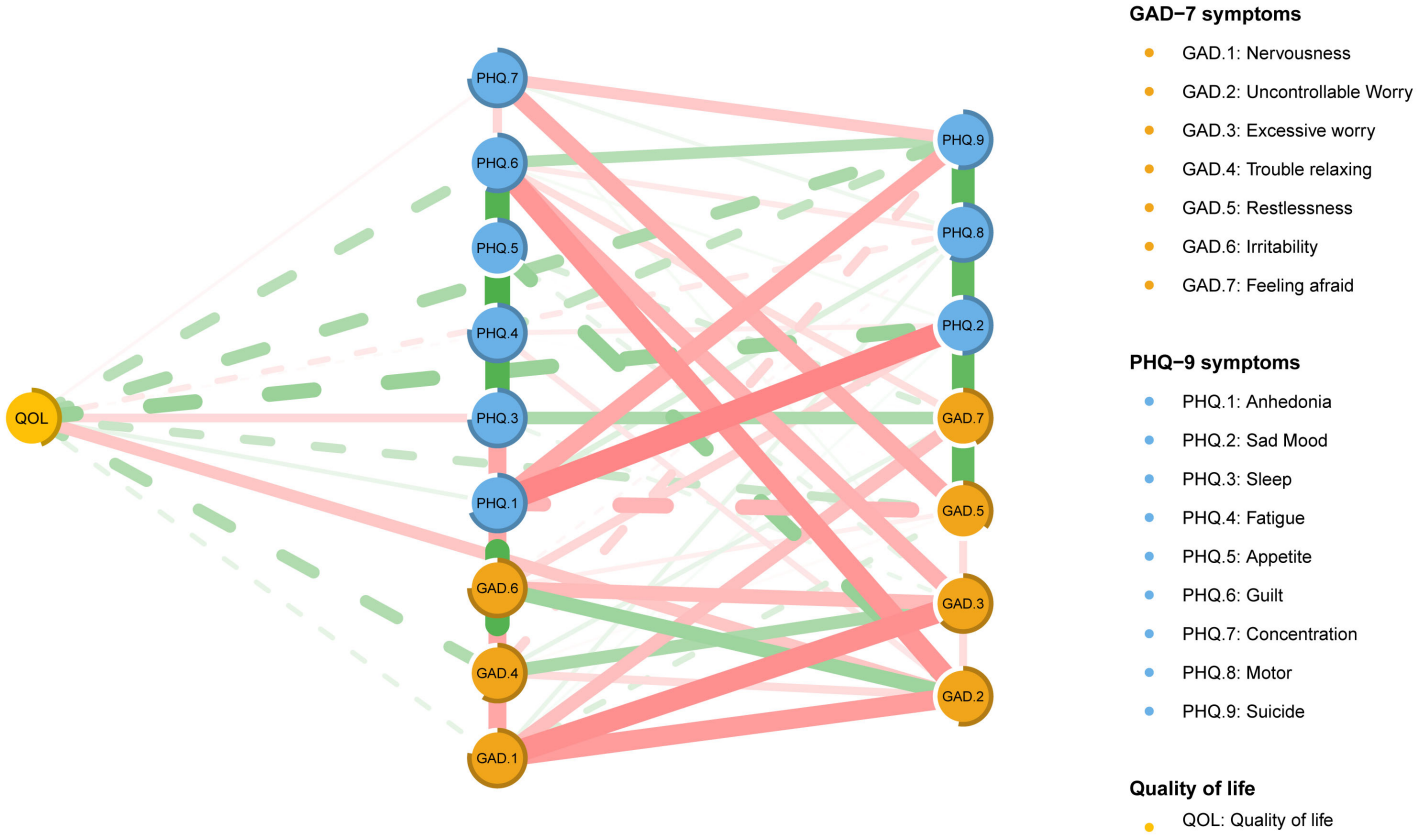
Network of conditional associations between depressive/anxiety symptoms and quality of life. This figure incorporates an external quality-of-life outcome node (QOL) into the depression–anxiety symptom network. The yellow node indicates quality of life, blue nodes indicate depressive symptoms, and orange nodes indicate anxiety symptoms. Edges represent undirected partial correlations between individual depressive/anxiety symptoms and quality of life after controlling for all other nodes in the network; they should not be interpreted as directional or causal effects. Green solid lines indicate positive associations and pink dashed lines indicate negative associations. Edge thickness reflects the strength of the conditional association. QOL, quality of life.

To examine independent statistical associations between symptoms and quality-of-life outcomes, external outcome flow networks were estimated (**Figure 4**). In the exploratory composite QOL model, the main symptom nodes showing positive partial correlation weights with QOL were GAD.4 (difficulty relaxing, weight = 0.210), GAD.6 (irritability, weight = 0.169), and PHQ.1 (anhedonia, weight = 0.160). The main symptom nodes showing negative partial correlation weights with QOL were PHQ.9 (suicidal ideation, weight = -0.141), GAD.7 (fear that something awful may happen, weight = -0.102), GAD.5 (restlessness, weight = -0.095), PHQ.8 (psychomotor changes, weight = -0.093), and PHQ.2 (depressed mood, weight = -0.072). Because higher WHOQOL-BREF scores indicate better QOL and higher symptom scores indicate greater symptom burden, we verified the direction of scaling before interpreting these signs. All bivariate Spearman correlations between symptom nodes and QOL outcomes were negative: composite QOL, ρ = −0.592 to −0.429; general health, ρ = −0.353 to −0.224; and overall QOL, ρ = −0.375 to −0.269. Thus, positive symptom-QOL partial edges in the composite flow network should not be interpreted as evidence that symptoms improve QOL; they most likely reflect conditional suppression arising from high symptom intercorrelations and adjustment for neighboring symptoms. In the supplementary analysis using “general health” as the external outcome (**Supplementary Figure 3**), the nodes with the strongest negative partial correlation weights were PHQ.2 (depressed mood, weight = -0.161), PHQ.9 (suicidal ideation, weight = -0.095), and GAD.5 (restlessness, weight = -0.088). PHQ.7 (concentration difficulties) showed a positive partial correlation with general health (weight = 0.091). In the supplementary analysis using “overall quality of life” as the external outcome (**Supplementary Figure 4**), the nodes with the strongest negative partial correlation weights were PHQ.7 (concentration difficulties, weight = -0.120) and PHQ.8 (psychomotor changes, weight = -0.114). After adjustment for age, sex, time since injury, injury segment, and AIS severity, the largest negative conditional associations for composite QOL were PHQ.2 (−0.205), GAD.4 (−0.173), PHQ.9 (−0.148), PHQ.8 (−0.108), and GAD.6 (−0.104). For general health, the largest negative associations were PHQ.2 (−0.171), GAD.4 (−0.098), GAD.5 (−0.092), PHQ.9 (−0.083), and PHQ.6 (−0.051); for overall QOL, the largest negative associations were PHQ.7 (−0.094), PHQ.8 (−0.063), PHQ.3 (−0.057), PHQ.5 (−0.057), and GAD.6 (−0.051). These results are summarized in **Supplementary Table 6**.

### 3.6 Subgroup network comparisons

Results of the Network Comparison Test (NCT) based on 1,000 permutations are shown in **Supplementary Figures 5, S6**. In the sex-stratified comparison, the male group (n = 262) and the female group (n = 54) did not differ significantly in network structure invariance (M = 0.328, P = 0.434) or global strength invariance (S = 0.743, P = 0.232). In the injury-severity comparison, the complete-injury group (n = 100) and the incomplete-injury group (n = 216) also did not differ significantly in network structure invariance (M = 0.264, P = 0.713) or global strength invariance (S = 0.067, P = 0.745). Given the relatively small female subgroup, these non-significant NCT results should be interpreted as absence of detected differences rather than evidence of full equivalence between sex subgroups.

## 4 Discussion

### 4.1 Main findings

Using symptom network analysis in 316 patients with subacute SCI, the present study characterized the topological structure of depression–anxiety comorbidity and further examined the independent statistical associations between key symptoms and quality-of-life outcomes. The overall network was dominated by positive partial correlation edges, indicating that depressive and anxiety symptoms remained closely interconnected even after controlling for other symptoms. Stronger edges were observed not only within the depressive symptom cluster but also between depressive and anxiety symptom communities, suggesting that the comorbidity structure was characterized by both within-community clustering and cross-community linkage. All findings should be understood as conditional association patterns in cross-sectional data, not as evidence of temporal ordering or directional symptom effects.

The prevalence results provide additional clinical context. When conventional ≥10 cutoffs were used, probable depression and anxiety rates were lower than pooled SCI estimates based on diagnostic or clinically significant symptom definitions; however, the ≥5 thresholds showed that mild-or-greater depressive and anxiety symptoms were common. This distinction is important because network analysis in the present study was designed to characterize symptom-level interrelations across the full range of symptom burden rather than only among patients meeting probable diagnostic cutoffs.

Centrality analyses showed that guilt, irritability, psychomotor changes, and concentration difficulties occupied prominent positions in the network, whereas depressed mood, concentration difficulties, irritability, and psychomotor changes exhibited relatively strong bridge effects. Flow network analyses further suggested that a subset of emotional symptoms showed relatively pronounced independent partial associations with composite quality of life, general health, and overall quality of life. In addition, network comparison tests indicated that the overall topology and global strength of the network did not differ significantly across sex or injury severity subgroups. Taken together, these findings suggest that depression–anxiety comorbidity in SCI may be better understood as an interacting system organized around a limited number of highly influential and highly connected symptoms, rather than a simple accumulation of individual complaints. The revised sensitivity analyses further suggest that the main symptom-level conclusions were not dependent on the very small numerical perturbation used in the primary estimation, although signs of specific partial edges, especially symptom-QOL edges, require cautious interpretation.

### 4.2 Network characteristics of core symptoms and their possible implications

Guilt (PHQ.6) ranked among the most central nodes in the EI analysis, suggesting that it may occupy an important position in the overall symptom system. In patients with SCI, guilt may be related to functional dependence, reduced self-care capacity, increased reliance on family caregivers, and negative self-appraisal associated with the loss of previous social roles. Prior work has shown that psychological adjustment after SCI is often accompanied by reduced self-worth and uncertainty about the future, factors that may render some patients more vulnerable to self-blame and feelings of guilt (21, 22). At the same time, the cross-sectional design means that the present findings cannot establish whether guilt temporally precedes other symptoms; rather, they indicate that guilt is located in a structurally important position within the network.

Irritability (GAD.6) also showed high EI and bEI values, indicating that it may be important both within the overall network and in linking depressive and anxiety symptom communities. Patients with SCI often experience chronic pain, sleep disturbance, restricted mobility, and sustained rehabilitation stress, all of which may be associated with irritability. Compared with the traditional emphasis on depressed mood or worry, the present findings suggest that irritability may occupy a particularly salient network position in the SCI context. This implies that irritability should not be regarded as a peripheral symptom in psychological assessment after SCI, but rather as a clinically relevant feature that deserves explicit attention.

Psychomotor changes (PHQ.8) and concentration difficulties (PHQ.7) also showed elevated centrality and bridge centrality, indicating that they are broadly connected within the network. Importantly, both symptoms have mixed somatic and psychological characteristics: on the one hand, they may reflect cognitive and motivational aspects of emotional disturbance; on the other hand, they may also be influenced by post-SCI fatigue, pain, sleep impairment, and neurological dysfunction. Accordingly, their high network positions should be interpreted cautiously and viewed as important manifestations within the emotional symptom system of subacute SCI rather than as purely specific psychopathological markers (12, 23).

The strongest edges observed in the network also have plausible symptom-content interpretations. The PHQ.9–PHQ.8 edge may reflect the co-occurrence of severe depressive risk markers with observable psychomotor slowing or agitation, whereas the PHQ.5–PHQ.4 edge reflects closely related somatic-energy symptoms, namely appetite change and fatigue. The PHQ.8–GAD.5 edge is particularly relevant to SCI because psychomotor change and restlessness share behavioral activation/retardation content and may be difficult to separate from injury-related motor limitations. These strong edges should therefore be viewed as clinically meaningful conditional associations, while still recognizing that they may partly reflect item-content overlap and the somatic context of SCI.

### 4.3 Bridge symptoms and the structure of depression–anxiety comorbidity

Bridge centrality analyses indicated that depressed mood, concentration difficulties, irritability, and psychomotor changes played relatively strong connective roles between the depression and anxiety communities. According to network theory, bridge symptoms may transmit activation across symptom clusters and thereby contribute to the maintenance of comorbidity (10, 24). In the current study, these high-bEI nodes suggest that depression and anxiety in SCI are not isolated symptom systems but are interconnected through a limited number of key symptoms. However, the data-driven community analysis did not recover a purely PHQ-versus-GAD partition and instead suggested mixed affective, cognitive, and somatic communities. Accordingly, the bridge findings are best interpreted as scale-defined depression-anxiety bridge centrality rather than as proof that the empirical network contains only two diagnostic communities.

Depressed mood, as one of the strongest bridge nodes, is consistent with its foundational role in affective disorders. At the same time, the finding that concentration difficulties, irritability, and psychomotor changes also showed high bridge centrality suggests that depression–anxiety comorbidity in SCI may involve intertwined affective, cognitive, and behavioral activation/retardation processes. In other words, in the SCI context, connections between depression and anxiety may not operate mainly through abstract emotional experience alone, but may also be mediated through problems in cognitive control, irritability, and psychomotor dysregulation. This pattern is not entirely identical to that seen in some studies of general populations, in which worry, nervousness, or sadness are often emphasized as dominant bridge symptoms, and may therefore reflect a degree of illness-context specificity in SCI.

Nevertheless, bridge centrality describes a node’s structural location within the network and should not be equated with a real-world unidirectional transmission pathway. The present findings are therefore better interpreted as identifying candidate bridge symptoms rather than proving that a given symptom causes the spread of another symptom cluster. Longitudinal or dynamic network designs will be needed to clarify whether these bridge nodes also play temporally predictive roles.

### 4.4 Independent associations between symptoms and quality-of-life outcomes

An important extension of the present study was the incorporation of quality-of-life indicators as external outcomes in flow network analyses. The results showed that composite quality of life, general health, and overall quality of life were not uniformly related to all symptoms; instead, they displayed differentiated patterns of independent conditional association. We verified that higher WHOQOL-BREF scores represented better QOL and that higher PHQ-9/GAD-7 scores represented greater symptom severity. Because all bivariate symptom-QOL correlations were negative, positive partial edges in the composite flow model should be interpreted as conditional suppression effects rather than as clinically intuitive evidence that greater symptoms are linked to better QOL.

In the composite QOL model, suicidal ideation, fear that something awful may happen, restlessness, psychomotor changes, and depressed mood showed relatively strong negative partial correlations with the outcome, suggesting that these symptoms remained associated with poorer quality of life even after adjustment for the broader emotional symptom network. By contrast, in the general-health model, depressed mood, suicidal ideation, and restlessness were more prominent, whereas in the overall-quality-of-life model, concentration difficulties and psychomotor changes showed stronger negative associations. These findings imply that different external outcomes may correspond to different symptom-level correlates and that symptom–outcome associations may be dimension-specific. Because the two WHOQOL-BREF global items showed inadequate internal consistency as a composite in this cohort, the item-specific general-health and overall-QOL findings should be given greater interpretive weight than the exploratory two-item composite.

This pattern has two implications. First, diminished subjective well-being in SCI may not simply reflect an elevation in total depression or anxiety scores, but may be more closely tied to a subset of specific symptoms. Second, different quality-of-life endpoints may be linked to different clinically salient symptoms. For example, outcomes more closely related to subjective health may be more strongly tied to depressed mood, suicidal ideation, and restlessness, whereas broader life-satisfaction outcomes may be more strongly tied to concentration problems and psychomotor abnormalities. These findings should be interpreted as evidence of independent statistical association rather than proof of deterministic causal effects of symptoms on quality of life (11–13).

### 4.5 Network invariance across subgroups

The study further examined whether the emotional symptom network differed by sex or injury severity. No statistically significant differences were observed in either network structure invariance or global strength invariance across these subgroup comparisons. This suggests that, in the present sample, the overall topological pattern of depression–anxiety symptom interactions was relatively consistent across these major clinical subgroups.

This finding has two implications. Statistically, the present data did not provide clear evidence that men and women or patients with complete versus incomplete injury have fundamentally different emotional comorbidity networks. Clinically, at least during the subacute stage, the interaction pattern of emotional symptoms in SCI may reflect a broadly shared psychological response to major functional loss and adaptation challenges rather than being determined primarily by sex or injury severity per se (3, 4, 20). At the same time, a non-significant NCT result should not be interpreted as proof of complete equivalence, especially given the relatively small number of female participants. Larger and multicenter samples will be needed to determine whether this apparent network invariance is stable.

### 4.6 Clinical implications

The present findings offer several potentially useful clues for psychological screening and rehabilitation management in SCI. First, reliance on PHQ-9 or GAD-7 total scores alone may be insufficient to identify the symptoms that occupy the most important positions in the comorbidity network. By contrast, identifying symptoms such as guilt, irritability, psychomotor changes, and concentration difficulties may help clinicians capture the current emotional risk structure of patients in a more nuanced way.

Second, the differentiated symptom–outcome associations observed across quality-of-life endpoints suggest that clinical goals for quality-of-life improvement may need to be specified more precisely. For example, when a patient mainly reports diminished subjective health, depressed mood, suicidal ideation, and restlessness may merit closer attention; when the main concern is low overall life satisfaction, concentration difficulties and psychomotor changes may be particularly relevant. These results are better understood as informing screening and prioritization rather than prescribing a single definitive treatment pathway. In clinical practice, prioritized symptoms such as irritability and psychomotor changes should be differentiated from overlapping injury-related manifestations through multidisciplinary assessment. For example, clinicians should examine temporal fluctuation, emotional triggers, patient-reported distress, medication changes, pain, spasticity, fatigue, sleep disturbance, and neurological motor limitations before attributing psychomotor or irritability symptoms solely to psychopathology. A symptom should be considered a psychological intervention target when it is persistent, distressing, contextually linked to mood or anxiety, and not fully explained by neurological impairment or rehabilitation-related somatic burden.

Finally, the present findings support the use of network analysis as a complement to conventional scale-based assessment for identifying key symptom patterns in SCI-related emotional disturbance. For rehabilitation physicians, psychologists, and nursing teams, a symptom-level perspective may help refine the prioritization of intervention targets.

### 4.7 Strengths and limitations

This study has several strengths. First, it focused specifically on the depression–anxiety comorbidity network in patients with subacute SCI, a clinically meaningful setting in rehabilitation medicine. Second, it examined not only core and bridge symptoms but also integrated quality-of-life outcomes into the network framework, thereby extending the clinical interpretability of the findings. Third, network accuracy, stability, and subgroup invariance were evaluated using multiple complementary procedures, including edge-weight bootstrapping, case-dropping bootstrap, and NCT. Fourth, all PHQ-9 and GAD-7 items were treated as four-category ordinal variables, and polychoric correlations were estimated where appropriate to account for their categorical nature (25).

Several limitations should also be acknowledged. First, the cross-sectional design can only reveal conditional dependence structures and cannot establish temporal ordering or causality among symptoms (7, 24). Second, all data were based on self-report measures and may therefore be influenced by reporting bias. Third, some symptom items—especially fatigue, sleep problems, and psychomotor changes—may partly overlap with somatic manifestations of SCI itself, so their network positions should be interpreted cautiously (22). Fourth, this was a single-center study, and the relatively small number of female participants may limit the generalizability of subgroup findings. Fifth, although the minimal random perturbation added to the data was used solely to ensure numerical stability, this technical procedure should still be transparently acknowledged when interpreting the results. Sixth, pain severity, psychotropic medication, and independent sleep-quality measures were not available, so the covariate-adjusted sensitivity analysis could adjust only for age, sex, time since injury, injury segment, and AIS severity. Seventh, the two WHOQOL-BREF global items did not form an internally consistent composite in this cohort; therefore, the item-specific QOL results should be prioritized and the composite QOL flow network should be considered exploratory. Finally, the male-dominant, single-center sample may reduce external validity and limit statistical power for sex-stratified NCT analyses, particularly in the female subgroup.

Overall, the present study suggests that depression–anxiety comorbidity in SCI is characterized by a relatively stable and hierarchically organized symptom structure. Guilt, irritability, psychomotor changes, and concentration difficulties occupied particularly important positions in the network, and some of these symptoms were also independently associated with quality-of-life outcomes. These findings support a symptom-level understanding of emotional problems in SCI and provide a basis for future longitudinal and intervention research.

## 5 Conclusion

Using symptom network analysis, this study systematically characterized the comorbidity structure of depressive and anxiety symptoms in patients with subacute SCI. The findings showed that the depression–anxiety network was dominated by positive conditional associations and organized around a limited number of key symptoms, particularly guilt, irritability, psychomotor changes, and concentration difficulties. Among these, depressed mood, concentration difficulties, irritability, and psychomotor changes also showed relatively strong bridge effects, suggesting that they may be especially important in linking the depressive and anxiety symptom communities.

Flow network analyses further indicated that some symptoms showed relatively pronounced independent statistical associations with composite quality of life, general health, and overall quality of life, implying that subjective health and quality-of-life impairment in SCI may be more closely related to certain specific symptoms than to total depression or anxiety burden alone. At the same time, network comparison tests detected no statistically significant differences in overall network structure or global strength across sex or injury severity subgroups. Because flow edges are undirected conditional associations, and because some positive partial symptom-QOL edges likely reflect suppression, these results should guide clinical hypothesis generation and screening rather than causal inference.

Overall, these findings suggest that emotional comorbidity in SCI may be conceptualized as an interacting system centered on a limited number of highly influential symptoms. Compared with relying solely on total questionnaire scores, identifying symptoms with elevated centrality, bridge effects, and stronger quality-of-life associations may better support refined psychological assessment and prioritization of intervention targets in SCI rehabilitation. Future longitudinal and multicenter studies are needed to determine the temporal stability and clinical implications of these key symptoms.

## Data Availability

The original contributions presented in the study are included in the article/**Supplementary Material**. Further inquiries can be directed to the corresponding authors.
