# Supplementary material for: Comorbidity network characteristics of depressive and anxiety symptoms and their associations with quality of life in patients with spinal cord injury: a cross-sectional study
Source: Front Psychiatry. 2026 Jul 24;17:1861743. doi: 10.3389/fpsyt.2026.1861743 (PMC13448545; doi:10.3389/fpsyt.2026.1861743)
Supplement: Supplementary file 1 [file Supplementaryfile1.docx]

# Supplementary Figure Legends

**Figure S1. Bootstrapped 95% confidence intervals of edge weights in the depression–anxiety network.**

Red line segments indicate the sample estimates of edge weights, black dotted lines indicate bootstrap means, and the gray regions indicate the distribution range and 95% confidence intervals of edge weights obtained from 1,000 nonparametric bootstrap resamples. Edges are ordered by estimated weight size. The close correspondence between sample estimates and bootstrap means suggests good stability of the edge-weight estimates.

**Figure S2. Bootstrapped significance test for differences in edge weights.**

This matrix presents the results of bootstrapped difference tests for edge weights based on 1,000 bootstrap resamples. Black cells indicate a statistically significant difference between the weights of two edges (p < 0.05), gray cells indicate non-significant differences, and the blue diagonal indicates a comparison of an edge with itself. This figure was used to evaluate whether the highest-weight edges were significantly stronger than other edges.

**Figure S3. Network of conditional associations between emotional symptoms and general health.**

**This figure shows undirected partial correlations between depressive/anxiety symptoms and the “General Health” node derived from the WHOQOL-BREF after controlling for all other symptom nodes in the network.** The yellow node indicates general health; green solid lines indicate positive associations and pink dashed lines indicate negative associations. Edge thickness reflects the strength of the independent conditional association. Abbreviation: G-Health, general health.

**Figure S4. Network of conditional associations between emotional symptoms and overall quality of life.**

**This figure shows undirected partial correlations between depressive/anxiety symptoms and the “Overall quality of life” node derived from the WHOQOL-BREF after controlling for all other symptom nodes in the network.** The yellow node indicates overall quality of life; green solid lines indicate positive associations and pink dashed lines indicate negative associations. Edge thickness reflects the strength of the conditional association. Abbreviation: O-QOL, overall quality of life.

**Figure S5. Network comparison test between male and female patients with spinal cord injury.**

The figure shows the estimated depression–anxiety symptom networks for female and male patients with spinal cord injury. Node and edge definitions are the same as in the main figures. Based on 1,000 permutation iterations, the network comparison test (NCT) showed no statistically significant differences between the two sex subgroups in either global strength invariance or network structure invariance.

**Figure S6. Network comparison test between complete and incomplete spinal cord injury subgroups.**

The figure shows the estimated depression–anxiety symptom networks for patients with complete and incomplete spinal cord injury. Node and edge definitions are the same as in the main figures. The network comparison test (NCT) showed no statistically significant differences between the two injury-severity subgroups in either global strength invariance or network structure invariance.

# Supplementary Tables

**Table S1. Descriptive statistics for PHQ-9 and GAD-7 items.**

| **Item** | **Mean (M)** | **SD** | **Skewness** | **Kurtosis** |
| --- | --- | --- | --- | --- |
| GAD.1 Nervousness | 0.63 | 0.62 | 0.68 | 0.70 |
| GAD.2 Uncontrollable worry | 0.41 | 0.59 | 1.30 | 1.57 |
| GAD.3 Excessive worry | 0.54 | 0.63 | 0.95 | 0.92 |
| GAD.4 Trouble relaxing | 0.49 | 0.64 | 1.23 | 1.62 |
| GAD.5 Restlessness | 0.25 | 0.48 | 1.92 | 3.98 |
| GAD.6 Irritability | 0.57 | 0.63 | 0.86 | 0.80 |
| GAD.7 Feeling afraid | 0.29 | 0.53 | 1.87 | 3.97 |
| PHQ.1 Anhedonia | 0.50 | 0.62 | 1.09 | 1.24 |
| PHQ.2 Sad mood | 0.43 | 0.57 | 1.13 | 1.35 |
| PHQ.3 Sleep problems | 0.59 | 0.71 | 1.14 | 1.17 |
| PHQ.4 Fatigue | 0.70 | 0.70 | 0.88 | 0.94 |
| PHQ.5 Appetite change | 0.52 | 0.67 | 1.23 | 1.45 |
| PHQ.6 Guilt/self-blame | 0.31 | 0.53 | 1.63 | 2.46 |
| PHQ.7 Concentration problems | 0.33 | 0.55 | 1.66 | 3.02 |
| PHQ.8 Psychomotor change | 0.24 | 0.48 | 2.05 | 4.52 |
| PHQ.9 Suicidal ideation | 0.14 | 0.39 | 3.16 | 12.26 |

*Note.* PHQ-9 = Patient Health Questionnaire-9; GAD-7 = Generalized Anxiety Disorder-7.

**Table S2. Edge-weight matrix of the depression–anxiety symptom network.**

Regularized partial correlation coefficients estimated by EBICglasso are displayed below. Diagonal elements are fixed at 0, and blank cells indicate that the upper triangle is omitted to avoid redundancy.

| **Item** | **GAD.1** | **GAD.2** | **GAD.3** | **GAD.4** | **GAD.5** | **GAD.6** | **GAD.7** | **PHQ.1** | **PHQ.2** | **PHQ.3** | **PHQ.4** | **PHQ.5** | **PHQ.6** | **PHQ.7** | **PHQ.8** | **PHQ.9** |
| --- | --- | --- | --- | --- | --- | --- | --- | --- | --- | --- | --- | --- | --- | --- | --- | --- |
| GAD.1 | 0.000 |  |  |  |  |  |  |  |  |  |  |  |  |  |  |  |
| GAD.2 | 0.293 | 0.000 |  |  |  |  |  |  |  |  |  |  |  |  |  |  |
| GAD.3 | 0.346 | 0.000 | 0.000 |  |  |  |  |  |  |  |  |  |  |  |  |  |
| GAD.4 | 0.110 | 0.103 | 0.208 | 0.000 |  |  |  |  |  |  |  |  |  |  |  |  |
| GAD.5 | -0.088 | 0.093 | 0.013 | 0.065 | 0.000 |  |  |  |  |  |  |  |  |  |  |  |
| GAD.6 | 0.000 | 0.231 | 0.218 | 0.212 | 0.063 | 0.000 |  |  |  |  |  |  |  |  |  |  |
| GAD.7 | 0.202 | 0.000 | 0.004 | 0.051 | 0.327 | 0.000 | 0.000 |  |  |  |  |  |  |  |  |  |
| PHQ.1 | 0.027 | 0.000 | 0.000 | 0.000 | -0.226 | 0.165 | 0.000 | 0.000 |  |  |  |  |  |  |  |  |
| PHQ.2 | 0.059 | 0.041 | 0.072 | 0.000 | 0.000 | 0.160 | 0.156 | 0.371 | 0.000 |  |  |  |  |  |  |  |
| PHQ.3 | 0.134 | 0.021 | -0.053 | 0.000 | 0.000 | -0.026 | 0.197 | 0.000 | 0.009 | 0.000 |  |  |  |  |  |  |
| PHQ.4 | -0.143 | 0.086 | 0.000 | 0.068 | 0.000 | 0.222 | 0.012 | 0.157 | 0.093 | 0.271 | 0.000 |  |  |  |  |  |
| PHQ.5 | 0.268 | -0.234 | -0.064 | 0.105 | -0.016 | -0.006 | -0.097 | 0.000 | 0.000 | 0.074 | 0.395 | 0.000 |  |  |  |  |
| PHQ.6 | -0.364 | 0.285 | 0.250 | 0.061 | 0.000 | 0.000 | 0.102 | 0.000 | 0.084 | 0.101 | -0.065 | 0.281 | 0.000 |  |  |  |
| PHQ.7 | 0.144 | 0.000 | 0.032 | 0.017 | 0.269 | -0.026 | 0.000 | 0.118 | 0.000 | 0.083 | 0.061 | 0.084 | 0.071 | 0.000 |  |  |
| PHQ.8 | 0.068 | 0.000 | 0.000 | 0.000 | 0.384 | -0.032 | 0.010 | 0.088 | 0.000 | 0.000 | 0.000 | 0.016 | 0.115 | 0.048 | 0.000 |  |
| PHQ.9 | 0.000 | 0.000 | -0.044 | -0.102 | 0.001 | -0.024 | 0.046 | 0.252 | -0.012 | 0.000 | -0.143 | 0.000 | 0.204 | 0.180 | 0.396 | 0.000 |

*Note.* Positive values indicate positive conditional associations, whereas negative values indicate negative conditional associations. Larger absolute values denote stronger independent links between symptom nodes.

**Table S3. Node predictability values (R2) for each PHQ-9 and GAD-7 symptom node.**

| **Node** | **Symptom** | **Predictability R2** |
| --- | --- | --- |
| GAD.1 | Nervousness | 0.616 |
| GAD.2 | Uncontrollable worry | 0.646 |
| GAD.3 | Excessive worry | 0.677 |
| GAD.4 | Difficulty relaxing | 0.666 |
| GAD.5 | Restlessness | 0.589 |
| GAD.6 | Irritability | 0.702 |
| GAD.7 | Feeling afraid | 0.608 |
| PHQ.1 | Anhedonia | 0.629 |
| PHQ.2 | Depressed mood | 0.655 |
| PHQ.3 | Sleep problems | 0.612 |
| PHQ.4 | Fatigue | 0.658 |
| PHQ.5 | Appetite changes | 0.591 |
| PHQ.6 | Guilt/self-blame | 0.622 |
| PHQ.7 | Concentration difficulties | 0.630 |
| PHQ.8 | Psychomotor changes | 0.598 |
| PHQ.9 | Suicidal ideation | 0.437 |

Note. Predictability represents the proportion of variance in each node explained by neighboring nodes in the estimated network.

**Table S4. Sensitivity analyses of centrality ranks, strongest edges, and suppression-related edges.**

| **Sensitivity model** | **Top EI nodes** | **Top bEI nodes** | **Strongest edges** | **PHQ.6–GAD.1** | **PHQ.9 EI (z/rank)** |
| --- | --- | --- | --- | --- | --- |
| Pearson, no jitter | PHQ.7, GAD.6, GAD.3, PHQ.8, GAD.4 | GAD.7, GAD.5, PHQ.2, PHQ.7, GAD.2 | GAD.5–PHQ.8 (0.314); PHQ.4–PHQ.5 (0.298); PHQ.1–PHQ.2 (0.278); PHQ.8–PHQ.9 (0.265); PHQ.3–PHQ.4 (0.261) | -0.148 | -2.934 |
| Spearman, no jitter | GAD.6, PHQ.8, PHQ.7, PHQ.6, PHQ.1 | GAD.7, PHQ.7, PHQ.8, PHQ.2, GAD.6 | PHQ.8–PHQ.9 (0.297); PHQ.3–PHQ.4 (0.283); PHQ.4–PHQ.5 (0.281); GAD.5–PHQ.8 (0.266); GAD.1–GAD.3 (0.246) | -0.166 | -2.992 |
| Polychoric, no jitter | PHQ.6, GAD.1, PHQ.4, GAD.5, GAD.6 | PHQ.6, PHQ.4, PHQ.2, GAD.7, GAD.5 | GAD.1–PHQ.6 (-0.682); PHQ.4–PHQ.5 (0.604); GAD.1–PHQ.5 (0.599); PHQ.5–PHQ.6 (0.580); GAD.1–GAD.2 (0.568) | -0.682 | -0.831 |
| EBIC γ = 0.25 and γ = 0.75 | Key high-ranking nodes overlapped with the primary network | PHQ.2, PHQ.7, GAD.6, and PHQ.8 remained clinically prominent | The five strongest edges in the primary model remained among the dominant edges | Negative in dense no-jitter checks; attenuated or shrunk in some sparse regularized checks; interpreted cautiously | Remained in the lower EI range; not interpreted as protective |

Note. No-jitter sensitivity analyses were performed without the Gaussian perturbation used in the primary estimation. EBIC tuning sensitivity is summarized qualitatively because edge density changed as expected with γ, while the principal clinical conclusions were unchanged.

**Table S5. Data-driven community-detection robustness check.**

| **Data-driven community-detection result** | **Nodes / values** |
| --- | --- |
| Algorithm and weighting | Greedy modularity community detection using absolute regularized EBICglasso edge weights; modularity = 0.188; adjusted Rand index vs. PHQ/GAD scale partition = -0.084. |
| Community 1 | GAD.1, GAD.2, GAD.3, GAD.4, PHQ.3, PHQ.4, PHQ.5, PHQ.6 |
| Community 2 | GAD.5, GAD.7, PHQ.7, PHQ.8, PHQ.9 |
| Community 3 | GAD.6, PHQ.1, PHQ.2 |
| Highest data-driven bridge nodes (signed between-community EI) | GAD.6 (0.832), GAD.7 (0.627), PHQ.7 (0.584), PHQ.6 (0.576), PHQ.2 (0.502) |
| Interpretation | The empirical communities were mixed rather than strictly PHQ vs. GAD; scale-defined bEI is therefore interpreted as a theory-driven PHQ-GAD comorbidity index. |

Note. Community detection was based on a greedy modularity algorithm applied to absolute regularized edge weights. The empirical communities were mixed rather than strictly PHQ versus GAD, supporting cautious interpretation of scale-defined bridge centrality.

**Table S6. Covariate-adjusted flow-network sensitivity analysis.**

| **QOL node** | **Largest negative conditional associations** | **Largest positive conditional associations** |
| --- | --- | --- |
| whoqol_global_total | PHQ.2 (-0.205); GAD.4 (-0.173); PHQ.9 (-0.148); PHQ.8 (-0.108); GAD.6 (-0.104) | GAD.7 (0.093); GAD.2 (0.022); GAD.3 (0.022); PHQ.1 (0.002) |
| whoqol_general_health | PHQ.2 (-0.171); GAD.4 (-0.098); GAD.5 (-0.092); PHQ.9 (-0.083); PHQ.6 (-0.051) | PHQ.7 (0.075); GAD.7 (0.041); GAD.3 (0.040); PHQ.5 (0.034); GAD.1 (0.013) |
| whoqol_overall_qol | PHQ.7 (-0.094); PHQ.8 (-0.063); PHQ.3 (-0.057); PHQ.5 (-0.057); GAD.6 (-0.051) | GAD.5 (0.029); PHQ.2 (0.027); GAD.7 (0.024); GAD.2 (0.006); PHQ.1 (0.003) |

Note. Variables were residualized for age, sex, time since injury, injury segment, and AIS severity before estimating partial associations.

**Table S7. Missingness, screening prevalence, and QOL scoring checks.**

| **Check** | **Result** |
| --- | --- |
| Item-level missing data | 0 missing values across all included variables |
| PHQ-9 ≥ 10 | 27 / 316 (8.5%) |
| GAD-7 ≥ 10 | 14 / 316 (4.4%) |
| PHQ-9 ≥ 5 | 111 / 316 (35.1%) |
| GAD-7 ≥ 5 | 103 / 316 (32.6%) |
| Comorbid PHQ-9 and GAD-7 ≥ 5 | 85 / 316 (26.9%) |
| WHOQOL-BREF global-item scaling | Higher scores indicate better QOL/general health; no reverse scoring was applied to these two items |
| Two-item QOL composite reliability | Pearson r = -0.397; Cronbach’s α = -1.318; item-specific analyses emphasized |
| SCI depression meta-analysis context | Prior meta-analytic literature reported a pooled depression estimate of approximately 22.2% after SCI; the PHQ-9 ≥10 rate in this cohort was lower, whereas the ≥5 burden showed that milder depressive symptoms were common. |
| SCI anxiety meta-analysis context | Prior SCI meta-analytic literature reported approximately 27% self-reported clinically significant anxiety; the GAD-7 ≥10 rate in this cohort was lower, whereas the5 burden was closer to broad screening-based caseness. |
